# Editorial: Understanding scleroderma: symptoms, causes, treatment options, and advanced diagnostic techniques

**DOI:** 10.3389/fimmu.2025.1716460

**Published:** 2025-10-07

**Authors:** Predrag Ostojic

**Affiliations:** Institute of Rheumatology, School of Medicine, University of Belgrade, Belgrade, Serbia

**Keywords:** systemic sclerosis, classification, adhesion molecules, integrins, lung cancer

Systemic sclerosis (SSc) is a chronic connective tissue disease, which is characterized by overproduction and deposition of collagen into the skin and internal organs (1). According to the extent and severity of skin fibrosis, patient can be classified into the limited and diffuse subset of the disease. Some patients have internal organ involvement without sclerodermatous skin changes. These patients are classified as heaving SSc sine scleroderma. However, there is a group of diseases that mimic scleroderma in their clinical presentation - these are scleroderma-like syndromes. Romanowska-Próchnicka et al. gave a comprehensive overview of such diseases, including rare conditions that share common features of SSc. This extensive group of diseases includes different subsets of localized scleroderma, inflammatory diseases (i.e. eosinophilic fasciitis, lichen sclerosus, POEMS syndrome, graft-versus-host disease), deposition disorders, drug-induced (iatrogenic) sclerosis, metabolic and paraneoplastic syndromes. The main features that distinguish scleroderma-like syndromes from SSc are the absence of Raynaud’s phenomenon, ANA and typical changes on capillaroscopy.

The etiology and pathogenesis of the disease is still not completely clarified. Vasculopathy, activation of the immune system and fibrosis are the three cornerstones of the disease. Endothelial damage and vascular dysfunction are considered as early, and in most cases, the first manifestations of the disease. Endothelial cells may be damaged by autoantibodies (i.e. anti-endothelial cell antibodies), viral agents or oxidative stress. SSc is characterized by increased levels of circulating cell adhesion molecules involved in endothelial dysfunction (2). Mangoni and Zinellu conducted a meta-analysis of 43 studies, investigating various types of circulating cell adhesion molecules in SSc patients, and found that Intercellular Adhesion Molecule 1 (ICAM-1), vascular cell adhesion molecule 1 (VCAM-1), Platelet Endothelial Cell Adhesion Molecule-1 (PECAM-1), E-selectin, and P-selectin are increased in patients with the disease. Moreover, an increased expression of adhesion molecules, such as Integrins, can be noticed on endothelial cells. In tissues integrins represent a morphological link between the extracellular matrix and intracellular cytoskeleton. There is a growing body of evidence in the literature that activation of integrins plays one of the key roles in the pathogenesis of SSc. Talins (talin1 and talin2 subtypes) and kindlins (kindlin 1,2,3 subtypes) are cytosolic proteins that directly interact with integrins and are essential in integrin activation and signaling (3). By binding to the cytosolic tail of integrin β subunits, talins and kindlins might activate integrins and promote cell migration and remodeling of the extracellular matrix. Xu et al. have found an increased expression of talin1 in skin lesions and serum of SSc patients, and identified the molecular mechanism of how talin1 leads to fibroblast activation and skin fibrosis. On the other hand, inhibition of talin1 demonstrates an anti-fibrotic effect. Therefore, talin1 appears to be a potent therapeutic target against skin fibrosis. Moreover, SSc is characterized by a significant up-regulation of vascular endothelial growth factor (VEGF), a pro-angiogenic factor, which contributes to angiogenesis and fibroblast activation as well (4). A meta-analysis of 42 studies by Zinellu and Mangoni indicates significantly higher serum concentration of VEGF in SSc patients compared to healthy subjects. Moreover, VEGF concentrations are found to be higher in patients with diffuse SSc, than in limited SSc. Concentration of VEGF did not differ among patients with or without digital ulcers, interstitial lung disease, telangiectasias, corticosteroids, immunosuppressors and vasodilators used to treat the disease. Beside up-regulated VEGF, SSc patients have a notably increased production of anti-angiogenic molecules such as pentraxin-3, endostatin, and angiostatin as well. Mangoni and Zinellu in another systematic review of 19 studies found that circulating endostatin concentrations were significantly higher in SSc patients than controls, as well as in patients with digital ulcers or pulmonary arterial hypertension (PAH). Endostatin is a circulating glycoprotein with anti-angiogenic effects, by blocking the binding of VEGF to its receptors, VEGFR-1 and VEGFR-2 (5). Moreover, it exerts significant anti-fibrotic effects, by downregulation of transforming growth factor β1 and blocking platelet-derived growth factor pathways. Finally, studies using proteomic analysis have reported that endostatin can significantly predict the clinical progression of SSc, supporting its role as a candidate biomarker in the clinical evaluation.

Certain blood cell types, particularly neutrophils, platelets, lymphocytes, and monocytes, have been shown to play an important role in the pathophysiology of SSc. In other immune-mediated inflammatory diseases (i.e. rheumatoid arthritis, psoriasis, systemic lupus erythematosis) a higher neutrophil-to-lymphocyte ratio (NLR), platelet-to-lymphocyte ratio (PLR), and monocyte-to-lymphocyte ratio (MLR) have been noticed, compared to healthy subjects (6–8). These parameters may successfully discriminate patients with active disease and patients in remission. Zinellu and Mangoni in a meta-analysis of 10 studies found that the NLR and the PLR are significantly higher in SSc patients than in healthy controls. Moreover, NLR, PLR and MLR are higher in SSc patients with interstitial lung disease (ILD), PAH and digital ulcers, compared to SSc patients without these complications. This meta-analysis shows that blood cell-derived indices of inflammation can be useful in the diagnosis of SSc and associated complications, monitoring of disease activity, and assessment of the effect of treatments.

Compared to the general population, patients with SSc more often suffer from malignant tumors (9). Lung cancer is the most prevalent tumor, followed by breast cancer. Risk factors for the occurrence of lung cancer in SSc patients are already identified and include presence of ILD, smoking, longer disease duration, anti-topo-I antibodies, a history of renal crisis, and male gender. Beside the disease itself, and the immunosuppressive medications used to treat it, both SSc and malignant diseases may share genetic background. Pan et al. found that the *PRKG2 (Protein Kinase CGMP-Dependent 2*) is one of the genes shared by SSc and lung cancer, affecting the proliferation and invasion of lung cancer cells.

## References

[1] Allanore Y Simms R Distler O Trojanowska M Pope J Denton CP Varga J. Systemic sclerosis. Nat Rev Dis Primers. (2015) 1:15002. doi: 10.1038/nrdp.2015.2, PMID: 27189141

[2] McEver RP. Selectins: initiators of leucocyte adhesion and signalling at the vascular wall. Cardiovasc Res. (2015) 107:331–9. doi: 10.1093/cvr/cvv154, PMID: 25994174 PMC4592324

[3] Tadokoro S Shattil SJ Eto K Tai V Liddington RC de Pereda JM. Talin binding to integrin beta tails: a final common step in integrin activation. Science. (2003) 302:103–6. doi: 10.1126/science.1086652, PMID: 14526080

[4] Maurer B Distler A Suliman YA Gay RE Michel BA Gay S. Vascular endothelial growth factor aggravates fibrosis and vasculopathy in experimental models of systemic sclerosis. Ann Rheum Dis. (2014) 73:1880–7. doi: 10.1136/annrheumdis-2013-203535, PMID: 23918036

[5] Kim YM Hwang S Kim YM Pyun BJ Kim TY Lee ST. Endostatin blocks vascular endothelial growth factor-mediated signaling via direct interaction with KDR/Flk-1. J Biol Chem. (2002) 277:27872–9. doi: 10.1074/jbc.M202771200, PMID: 12029087

[6] Wang L Wang C Jia X Yang M Yu J. Relationship between neutrophil-to-lymphocyte ratio and systemic lupus erythematosus: A meta-analysis. Clinics (Sao Paulo). (2020) 75:e1450. doi: 10.6061/clinics/2020/e1450, PMID: 32321113 PMC7153360

[7] Zinellu A Mangoni AA. Neutrophil-to-lymphocyte and platelet-to-lymphocyte ratio and disease activity in rheumatoid arthritis: A systematic review and meta-analysis. Eur J Clin Invest. (2023) 53:e13877. doi: 10.1111/eci.13877, PMID: 36121342

[8] Paliogiannis P Satta R Deligia G Farina G Bassu S Mangoni AA. Associations between the neutrophil-to-lymphocyte and the platelet-to-lymphocyte ratios and the presence and severity of psoriasis: a systematic review and meta-analysis. Clin Exp Med. (2019) 19:37–45. doi: 10.1007/s10238-018-0538-x, PMID: 30478648

[9] Lepri G Catalano M Bellando-Randone S Pillozzi S Giommoni E Giorgione R. Systemic sclerosis association with Malignancy. Clin Rev Allergy Immunol. (2022) 63:398–416. doi: 10.1007/s12016-022-08930-4, PMID: 36121543 PMC9674744

